# The Use of Epidermal Growth Factor Receptor Type 2-Targeting Tyrosine Kinase Inhibitors in the Management of Epidermal Growth Factor Receptor Type 2-Positive Gastric Cancer: A Narrative Review

**DOI:** 10.7759/cureus.6295

**Published:** 2019-12-05

**Authors:** Asim M AlMazmomy, Majed M Al-Hayani, Mohammed Alomari, Abdulrahman G Bazi

**Affiliations:** 1 Surgery, College of Medicine King Abdulaziz University, Rabigh, SAU; 2 Neurology, College of Medicine King Abdulaziz University, Rabigh, SAU; 3 Pediatrics, College of Medicine King Abdulaziz University, Rabigh, SAU; 4 Internal Medicine, College of Medicine King Abdulaziz University, Rabigh, SAU

**Keywords:** gastric cancer, gastroesophageal junction cancer, human epidermal growth factor receptor 2, tyrosine kinase inhibitors

## Abstract

Gastric cancer (GC), including gastroesophageal junction cancer (GEJC), continues to be one of the most frequently diagnosed neoplasms globally. Moreover, GC/GEJC is a principal cause of neoplasm-related fatalities. Early-stage GC/GEJC has a favorable five-year overall survival (OS) rate with surgical resection. However, the vast majority of patients present with advanced inoperable or metastatic disease with a very unfavorable five-year OS rate. Such patients are left with very limited therapeutic options, such as systemic chemotherapy, targeted therapy, and immunotherapy, all of which can be performed as monotherapy or in various combinations. The molecular profiling of GC has revealed several personalized therapeutic vulnerabilities, one of which is the expression of epidermal growth factor receptor type 2 (EGFR2, also known as HER2). HER2 overexpression or amplification is present in a fair subset of patients with GC/GEJC and has been shown to correlate with poor clinicopathological prognostic outcomes. Generally, treatment schemes to tackle HER2 in HER2-positive GC/GEJC comprise the use of anti-HER2 monoclonal antibodies or HER2-targeting tyrosine kinase inhibitors (TKIs). In this study, we engage in a narrative review of the available phase II and III literature on the efficacy and safety of HER2-targeting TKIs in the management of HER2-positive GC/GEJC.

## Introduction and background

Gastric cancer (GC), including gastroesophageal junction cancer (GEJC), is the fifth most commonly diagnosed malignancy and the third leading cause of malignancy-related mortality worldwide [1]. Generally, its incidence is two-fold higher in males than in females [1]. Its incidence frequency is largely concentrated in East Asia, particularly Japan, China, and Korea [1]. The five-year overall survival (OS) rates for GC/GEJC patients with local (stage I-II), regional (stage III), and distant (stage IV) diseases are 68%, 31%, and 5%, respectively [2]. Surgical resection is the curative standard of care in patients with early-stage disease. Unfortunately, most patients seek clinical attention when the disease is in an advanced stage in which curative surgical resection is not technically feasible [3]. Patients with primary advanced inoperable, recurrent, or metastatic GC/GEJC usually receive systemic chemotherapy. Even though systemic chemotherapy has shown substantially improved progression-free survival (PFS) and OS rates in patients with GC/GEJC when compared to supportive care, the five-year OS rate still does not extend beyond 20-30% [4,5]. One potential reason for the reduced OS is the inherent heterogeneity of GC/GEJC at the clinical, histological, and molecular levels, which mandates a pressing need to devise personalized treatment approaches in terms of targeted therapy [6].

In line with translating GC/GEJC genomics into targeted therapies, molecular profiling analysis has identified epidermal growth factor receptor type 2 (EGFR2, also known as HER2) as a therapeutic vulnerability [7]. HER2 is one of the four members of the EGFR family of receptor tyrosine kinases. At the cellular level, HER2 is implicated in the regulation of cell proliferation, migration, differentiation, and adhesion. HER2 does not bind to specific ligands, and it is activated through hetero- or homo-dimerization with the other EGFR members, leading to aberrant activation of important oncogenic signaling pathways, such as phosphatidylinositol three-kinase/protein kinase B/mammalian target of rapamycin (PI3K/AKT/mTOR) [8,9]. HER2 positivity can be established through immunohistochemistry (IHC) and/or fluorescence in situ hybridization (FISH), and it is seen in at least more than 15% of patients with GC/GEJC [10,11]. At the clinical level, HER2 expression is correlated with poor clinicopathological prognostic outcomes, such as old age, advanced-stage disease, large tumor size, high tumor grade, lymphovascular space invasion, and poor survival rates [12-15]. Thus, targeting HER2 in HER2-positive GC/GECJ is a plausible therapeutic approach. Of note, HER2 overexpression or amplification in GC/GEJC denotes poor prognosis even in the early stages of GC/GEJC [16]. Therapeutic strategies to target HER2 in HER2-positive GC/GEJC include anti-HER2 monoclonal antibodies and HER2-targeting tyrosine kinase inhibitors (TKIs). The primary aim of this study is to engage in a narrative review of the available phase II and III literature on the efficacy and safety of HER2-targeting TKIs in the management of HER2-positive GC/GEJC. 

## Review

Literature search strategy

We screened the PubMed® database for the period from January 1, 2000 to October 1, 2019 by using the following keywords: “gastric cancer” OR “gastroesophageal junction cancer” OR “HER2 positive” OR “EGFR2 positive” OR “tyrosine kinase inhibitor”. Additional references from published articles were also manually screened for potential inclusion in the study analysis. The study inclusion criteria included: (1) studies published in the English language, (2) patients diagnosed with HER2-positive GC/GEJC, (3) studies reporting completed phase II or III trials, and (iv) studies reporting the efficacy and/or safety of the HER2-targeting TKIs lapatinib, afatinib, dacomitinib, and neratinib. For each study included in the review, the following details were also reviewed subject to availability: the first author, year of publication, trial type, trial identification number, study sample size, efficacy, toxicity, duration of follow-up, survival outcomes, and conclusions.

Lapatinib

Lapatinib is a dual small-molecule TKI which blocks EGFR type 1 (EGFR1, also known as HER1) and HER2 tyrosine kinase activities, resulting in the inhibition of intracellular signaling and suppressing of tumor proliferation [17]. Preclinical studies have demonstrated the in vitro and in vivo efficacy of lapatinib in various HER2-positive GC cells [18]. An earlier study by Iqbal et al. (NCT00103324, Southwest Oncology Group study S0413: a phase II trial) evaluated frontline lapatinib in 33 chemo-naïve patients with advanced or metastatic GC [19]. Patients were divided into two groups according to the median HER2 gene expression level: high (n = 16) and low (n = 17) median HER2 gene expression. An exploratory analysis showed that patients with high median HER2 gene expression had a statistically significant improved median OS than patients with low HER2 gene expression (6.8 vs. 3.1 months; p: 0.0031).

In 2014, Satoh et al. (NCT00486954: a phase III trial) examined the efficacy and safety of second-line lapatinib plus paclitaxel (n = 132) versus paclitaxel alone (n = 129) in 261 Asian patients with HER2-amplified GC [20]. The lapatinib-plus-paclitaxel group achieved a higher median OS (11 vs. 8.9 months, p: 0.1044), median PFS (5.4 vs. 4.4 months, p: 0.2441), median time-to-progression (TTP: 5.5 vs. 4.4 months, p: 0.2163), and overall response rate (ORR: 27 vs. 9%, p<0.001) than the paclitaxel-alone group. Nevertheless, the clinical improvements in OS, PFS, and TTP achieved by the lapatinib-plus-paclitaxel group were not statistically significant. An exploratory analysis demonstrated that Chinese nationality (as compared to Japanese nationality) and high HER2 IHC positivity (IHC3+ as compared to IHC0/IHC1+/IHC2+) were associated with favorable survival benefits in terms of OS and PFS (all p: <0.05). Treatment-related side effects of any grade were comparable between both groups (100 vs. 98%, respectively), and most of them were grade-I/-II adverse events. The study concluded that the lapatinib-plus-paclitaxel regimen was not associated with substantially advantageous survival benefits when compared to paclitaxel alone in patients with HER2-amplified GC. Nevertheless, Chinese nationality and HER2 positivity were correlated with better clinical efficacy.

In 2015, Lorenzen et al. (NCT01145404: a phase II trial) investigated the clinical utility and toxicity of second-line lapatinib plus capecitabine (n = 18) versus lapatinib alone (n = 19) in 37 patients with HER2-positive GC [21]. More than half of the patients in both groups had previously received an anti-HER2 monoclonal antibody trastuzumab therapy (67 vs. 63%, respectively). ORR was achieved in two patients (11.1%) in the lapatinib-plus-capecitabine group, both of which were partial responses. On the other hand, there was no ORR from the lapatinib-alone group (0%). Median TTP was higher in the lapatinib-plus-capecitabine group than in the lapatinib-alone group (83 vs. 42 days). The study was terminated too early due to ineffectiveness. The secondary endpoints of the study of median PFS and OS were largely comparable in both groups (PFS: 47 vs. 41 days; OS: median not reached vs. 142 days). The frequency of treatment-related side effects of any grade (100 vs. 90%) or ≥grade-III (55.6 vs. 52.6%, respectively) were similar between both groups. However, the incidence of diarrhea was more frequent in the lapatinib-plus-capecitabine group than in the lapatinib-alone group (61 vs. 26%). The study concluded that second-line lapatinib plus capecitabine was not clinically effective in previously treated patients with HER2-positive GC.

In 2016, Hecht et al. (NCT00680901: a phase III trial) gauged the therapeutic benefits and safety of frontline lapatinib plus capecitabine plus oxaliplatin (lapatinib group, n = 272) versus placebo plus capecitabine plus oxaliplatin (placebo group, n = 273) in 545 previously untreated patients with advanced or metastatic HER2-positive GC/GEJC [22]. Median OS (12.2 vs. 10.5 months, p: 0.3492), median PFS (6 vs. 5.4 months, p: 0.0381) and ORR (53 vs. 39%, p: 0.0031) were higher for the lapatinib group. Nevertheless, the clinical improvement in OS incurred by the lapatinib group was not statistically significant. An exploratory analysis exhibited no statistically significant association between HER2 IHC status and survival. However, Asian patients in the lapatinib group had superior median OS than the placebo group (16.5 vs. 10.9 months, p: 0.0261), and this advantageous correlation was demonstrated in a follow-up exploratory analysis as well [23]. Despite modest improvements in OS, PFS, and ORR, the NCT00680901 study concluded that the incorporation of lapatinib to capecitabine plus oxaliplatin did not yield any improved OS benefits in previously untreated patients with advanced or metastatic HER2-positive GC/GEJC.

In 2018, Moehler et al. (NCT01123473: a phase II trial) examined the efficacy of frontline lapatinib plus chemotherapy (lapatinib group, n = 5) versus placebo plus chemotherapy (placebo group, n = 5) in ten patients with metastatic HER2-positive GC [24]. HER2 positivity was confirmed by IHC plus FISH (stratum one) or IHC only (stratum two). Both strata represented the cohort of patients with HER2-positive status in the study. Options for chemotherapy were investigator-chosen and included epirubicin plus cisplatin plus fluorouracil (ECF), or epirubicin plus cisplatin plus capecitabine (ECX). ORR was not substantially different; two patients in the lapatinib group and one patient in the placebo group achieved partial responses. No survival data of PFS and OS were reported. Overall, the study concluded that lapatinib with chemotherapy (ECF or ECX) was not associated with appreciable efficacy in patients with metastatic HER2-positive GC. 

Another phase II trial that investigated the efficacy of neoadjuvant lapatinib plus capecitabine plus oxaliplatin in HER2-positive GC patients with liver metastasis has been undertaken (NCT02015169) [25]. However, the results have not yet been made public.

Afatinib

Afatinib is an irreversible nonspecific small-molecule TKI of the EGFR family. It is approved by the Food and Drug Administration (FDA) as a frontline treatment in patients with activating EGFR mutation-positive non-small-cell lung cancer. Afatinib has been shown to exhibit antitumor activities against HER2-positive GC cell lines through the downregulation of HER receptor tyrosine kinases as well as downstream kinase activation [26].

So far, there have been no published phase II or III trials of afatinib in the management of HER2-positive GC/GEJC. Nevertheless, there are two phase II trials currently being conducted to assess the efficacy of afatinib plus paclitaxel as a second-line regimen in trastuzumab-refractory patients with GC/GEJC (NCT01522768 and NCT02501603) [27,28]. Furthermore, there is a completed phase II trial that gauged the efficacy of frontline afatinib plus cisplatin plus fluorouracil in patients with inoperable GC. However, the results are still being analyzed and not yet published (NCT01743365) [29].

Dacomitinib

Dacomitinib, also recognized as PF-00299804, is a second-generation small-molecule pan-inhibitor of the EGFR family of receptor tyrosine kinases [30]. An earlier preclinical study has reported the anticancer ability of dacomitinib as a monotherapy as well as in combination with chemotherapy/targeted therapy in HER2-positive GC cell lines [31].

In 2016, Oh et al. (NCT01152853: a phase II trial) examined the efficacy, toxicity profile, and potential biomarkers of second-line dacomitinib in 27 pretreated patients with HER2-positive GC [32]. Specifically, seven patients (25.9%) had received prior anti-HER2 treatments. Overall, two (7.4%) and nine (33.3%) patients attained partial responses and stable disease, respectively, thus yielding a disease-control rate of 40.7% (partial responses plus stable disease). The median OS, median PFS, and 4-month PFS rate were 7.1 months, 2.1 months, and 22.2%, respectively. Slightly less than half of the patients (n = 11, 40.7%) exhibited some extent of tumor reduction. The vast majority of drug-related side effects were mild grade-I/-II adverse events, such as fatigue, skin rash, and diarrhea. An exploratory analysis of biomarkers showed that higher serum levels of HER2 extracellular domain and lower levels of soluble E-cadherin as positive prognostic markers of improved efficacy. The study showed that dacomitinib as a second-line agent was associated with acceptable, although not substantial, therapeutic benefits and endurable toxicity profile in previously treated patients with HER2-positive GC. There are no ongoing phase II or III clinical trials about dacomitinib in the management of patients with HER2-positive GC/GEJC.

Varlitinib

Varlitinib, also recognized as ASLAN001 and ARRY-334543, is a reversible small-molecule TKI of the EGFR family [10].

So far, there are no published phase II or III trials of varlitinib in the management of HER2-positive GC/GEJC. Nevertheless, a phase II trial explored the efficacy of varlitinib in patients with recurrent or metastatic GC. The tumors in these patients were either HER2-amplified or coexpressing HER1 and HER2. However, the results are not yet disseminated (NCT01614522) [33]. On the other hand, there is an ongoing phase II-III trial (NCT03130790) that attempts to explore the therapeutic efficacy and safety of frontline varlitinib plus folinic acid-leucovorin-fluorouracil-oxaliplatin (mFOLFOX6, interventional group) versus placebo plus mFOLFOX6 (placebo group) in patients with chemo-naïve advanced or metastatic HER1- and HER2-positive coexpressing GC [34].

Neratinib

Neratinib, also recognized as HKI-272, is a reversible small-molecule TKI of the EGFR family [10]. A preclinical study showed that neratinib exhibited therapeutic benefits in HER2-positive GC cells [9].

In 2018, Hayman et al. (NCT01953926: a phase II trial) examined the efficacy of neratinib in five patients with HER2-positive GC/GEJC. The results were largely disappointing as no patient achieved ORR and the median PFS achieved was 1.7 months. The study concluded that neratinib did not seem to offer clinical and survival benefits in patients with HER2-positive GC/GEJC. There are no ongoing phase II or III clinical trials about neratinib in the management of patients with HER2-positive GC/GEJC.

Discussion

The primary goal of molecular profiling and subclassification of GC/GEJC into subtypes is to facilitate the optimal selection of patients in whom molecular targeted therapies can achieve optimum results [6]. HER2 positivity or amplification has been identified in at least a quarter of patients with GC/GEJC [10]. Therapeutic approaches to target HER2 in HER2-positive GC/GEJC include anti-HER2 monoclonal antibodies and HER2-targeting TKIs. The similarities, differences, advantages, and disadvantages of each approach are documented in the literature [36]. HER2-targeting TKIs exhibit their antitumor activities by binding to the adenosine triphosphate (ATP)-binding site of the receptor’s intracellular domain, resulting in the inhibition of intracellular signaling and suppressing of tumor proliferation [36]. In this study, we aimed to review the efficacy of HER2-targeting TKIs employed in the management of patients with HER2-positive GC/GEJC.

Table 1 outlines a summary of phase II and III trials that explored HER2-targeting TKIs in the management of patients with HER2-positive GC/GEJC. Collectively, there were six published phase II and III clinical trials: two phase III trials and four phase II trials [20,21,22,24,32,35]. Among them, only two studies included sample sizes of more than 100 patients [20,22]. The setting of HER2-targeting TKI was first-line in two studies, second-line in three studies, and mixed (first-line and second-line) in one study [20,21,32,22,24,35]. Lapatinib was the most commonly reported HER2-targeting TKI (n = 4). Lapatinib monotherapy in 19 patients was not associated with favorable survival benefits, in terms of median PFS (1.5 months) and OS (5.1 months) [21]. The combination of lapatinib plus chemotherapy in 422 patients was associated with a mean PFS of 4.4 months (range: 1.7-6 months) and a mean OS of 11.6 months (range: 11-12.2 months. In one study, the median OS was not reached, and the OS was not reported in another one) [20-22,24]. In summary, the overall clinical and survival benefits of HER2-targeting TKIs for lapatinib, dacomitinib, and neratinib were discouraging and not superior to standard-of-care chemotherapy or placebo.

**Table 1:** A summary of the completed phase II and III trials utilizing epidermal growth factor receptor type two (HER2)-targeting tyrosine kinase inhibitors in the management of HER2-positive gastric cancer and gastroesophageal cancer ECF: epirubicin plus cisplatin plus fluorouracil; ECX: epirubicin plus cisplatin plus capecitabine; ID: identifier; NCT: national clinical trial; NR: not reported; ORR: overall response rate; OS: overall survival; PFS: progression-free survival; TTP: time-to-progression

Ref.	First author	Year	Phase	Clinical trial ID	Setting	Sample size	Regimen	ORR, %	TTP, months	PFS, months	OS, months	Overall results
[20]	Satoh	2014	III	NCT00486954	Second-line	132	Lapatinib + paclitaxel	27	5.5	5.4	11	Negative
						129	Paclitaxel	9	4.4	4.4	8.9	
[21]	Lorenzen	2015	II	NCT01145404	Second-line	18	Lapatinib + capecitabine	11.1	2.9	1.7	Not reached	Negative
						19	Lapatinib	0	1.5	1.5	5.1	
[22]	Hecht	2016	III	NCT00680901	First-line	272	Lapatinib + capecitabine + oxaliplatin	53	NR	6	12.2	Negative
						487	Placebo + capecitabine + oxaliplatin	39	NR	5.4	10.5	
[24]	Moehler	2018	II	NCT01123473	First-line	5	Lapatinib + ECF/ECX	40	NR	NR	NR	Negative
						5	Placebo + ECF/ECX	20	NR	NR	NR	
[32]	Oh	2016	II	NCT01152853	Second-line	27	Dacomitinib	7.4	NR	2.1	7.1	Negative
[35]	Hayman	2018	II	NCT01953926	Mixed	5	Neratinib	0	NR	1.7	NR	Negative

Even though several HER2-targeting TKIs have been developed, their overall therapeutic/prognostic impact on patients has not changed much over time [6]. This can be attributed to the inter- and intra-tumor heterogeneity of HER2-positive GC/GEJC [6]. Moreover, there is an urgent need to identify biomarkers of sensitivity and resistance for these HER2-targeting TKIs in GC/GEJC. Several preclinical studies have identified various biomarkers that confer resistance to lapatinib and afatinib in HER2-positive GC cell lines in vitro and in vivo [37-40,41]. However, these biomarkers have to be validated in human clinical trial studies. In addition, HER2-positive GC patients who were previously treated with anti-HER2 therapy should be inspected for HER2-positive status before the administration of second-line anti-HER2 TKIs. This is because a subset of relapsed patients may undergo conversion from HER2-positive to HER2-negative status [42]. Moreover, it should be noted that the tumor biology of HER2-positive GC/GEJC and HER2-positive breast cancer is not the same, and this may dramatically impact anticancer efficacy. For example, while neratinib offered survival benefits in HER2-positive breast cancer [43], neratinib was largely therapeutically not beneficial in patients with HER2-positive GC/GEJC [35].

Table 2 summarizes the in-progress phase II and III clinical trials of HER2-targeting TKIs in patients with HER2-positive GC/GEJC.

**Table 2:** A summary of completed (but not published) and ongoing phase II/III trials that use epidermal growth factor receptor type two (HER2)-targeting tyrosine kinase inhibitors in the management of HER2-positive gastric cancer and gastroesophageal cancer GC: gastric cancer; GEJC: gastroesophageal junction cancer; HER1: epidermal growth factor receptor type one; HER2: epidermal growth factor receptor type two; mFOLFOX6: modified folinic acid plus 5-fluorouracil plus oxaliplatin; NCT: national clinical trial; Ref: reference

Ref	NCT	Phase	Study design	Status
[25]	NCT02015169	II	Neoadjuvant lapatinib plus capecitabine plus oxaliplatin in HER2-positive GC patients with liver metastasis	Completed, not published
[27]	NCT01522768	II	Afatinib plus paclitaxel as second-line therapy in patients with advanced HER2-positive trastuzumab-refractory advanced GEJC	Ongoing
[28]	NCT02501603	II	Afatinib plus paclitaxel as second-line therapy in patients with advanced HER2-positive GC	Ongoing
[29]	NCT01743365	II	Frontline afatinib plus cisplatin plus 5-fluorouracil in patients with inoperable GC	Completed, not published
[33]	NCT01614522	II	Varlitinib in patients with recurrent/metastatic GC whose tumors are either HER2-amplified or coexpressing HER1 and HER2	Completed, not published
[34]	NCT03130790	II-III	Frontline varlitinib plus mFOLFOX6 vs. placebo plus mFOLFOX6 in chemo-naïve patients with advanced/metastatic HER1- and HER2-positive coexpressing GC	Ongoing

Our study has two primary limitations that should be acknowledged. First, limiting our literature search to the PubMed® database only (as opposed to other databases) and opting for a narrative review (as opposed to a systematic review) research design might have resulted in a failure to include all potential published phase II and III trials. Second, the very few numbers of the published phase II and III trials (n = 5), the heterogeneity of study settings, and the small sample sizes of patients made it difficult to conduct a meta-analysis and deduce concrete conclusions about the utility of HER2-targeting TKIs in the management of patients with HER2-positive GC/GEJC.

## Conclusions

In patients with HER2-positive GC/GEJC, molecular targeted therapy using current HER2-targeting TKIs (lapatinib, afatinib, dacomitinib, and neratinib) largely does not yield substantial clinical and survival benefits. Thus, the utility of current HER2-targeting TKIs in the management of HER2-positive GC/GEJC is questionable. As it stands, anti-HER2 monoclonal antibodies are preferred over HER2-targeting TKIs in the management of HER2-positive GC/GEJC. Prospective research may include synthesis of new HER2-targeting TKIs or identification of synergistic combinations utilizing old HER2-targeting TKIs plus/minus cytotoxic chemotherapy plus/minus additional molecular targeted therapy plus/minus immunotherapy. The ultimate goal should be to develop HER2-targeting regimens that are capable of offering maximum therapeutic efficacy with minimum side effects.
